# The Book World of Medicine and Science

**Published:** 1897-12-25

**Authors:** 


### The Book World of Medicine and Science.

The Diseases of Women: A Handbook for Students and Practitioners. By J. Bland Sutton, F.R.C.S.Eng., and Arthur E. Giles, M.D., B.Sc.Lond., F.R.C.S. Edin. (London: The Rebman Publishing Company. 1897. Pp. 436. Illustrations 115. Price 14s.)

This is a book which we can with great confidence recommend. In it the student will find the facts of gynæcology arranged in that orderly and systematic manner which is necessary for those who take up the subject for examination purposes, while the practitioner will find scattered over its pages many practical suggestions which will be of the utmost service to him in his daily round. The work consists of forty-seven chapters. The first three are devoted to a consideration of the anatomy, physiology, and methods of examination of the points involved; then come three on malformations and their consequences; the rest of the volume being devoted to the various diseases, their diagnosis and treatment. The treatment of diseases of women has become so largely surgical that we need not be surprised to find a considerable number of chapters given up to a description of gynæcological operations. This is the part of the book we like the best, but so evenly balanced is the distribution of space to the different subjects, and so careful have the authors been to hold in restraint all tendency to hobby-riding, that in saying this we would like specially to guard ourselves from being thought to mean that any undue prominence has been given to the surgical side. The description of the operations are short and to the point, the teaching being definite and showing practical experience. Lists of instruments which are required in all operations are given in two groups, according as they are vaginal or abdominal; and under the head of each operation the necessary supplementing instruments are mentioned. This is a detail which will be found very useful by those going in for examination, and by no means unserviceable even by their seniors. Among other matters, we are told that a new fish kettle makes an excellent steriliser. The crutch is an instrument which very properly receives high praise, and it is laid down as a general principle that "in all vaginal operations the patient lies on her back." This is good; for an operation, however slight, which is done in the lithotomy position is at any rate seriously entertained. The use of the écraseur for the removal of uterine polypi is thus spoken of: "Some gynæcologists employ an antiquated instrument called the 'écraseur' to divide the stalks of pedunculated submucous myomata. In a few years it is to be hoped that this instrument will only be seen in museums." In perineorrhaphy, and also in trachelorrhaphy, the author advises the use of "shot and coil" for fastening the sutures, a method which certainly has great advantages over the older plan by an ordinary split shot. They describe it as follows: "The mode of securing the sutures is of some importance. It is usual to run a small coil of silver wire along the two projecting ends of the silkworm gut or wire, and then draw the ends through a perforated shot; the parts are then drawn sufficiently tight and the shot secured by squeezing it with the shot-compressor." The shot is easily cut away, when the coil slips off and exposes the end of the suture, which can be withdrawn. The following dictum is definite enough, and may prevent many operative disasters: "Any sponge which has been in contact with a septic wound or pus should be promptly cast into the fire." For cleansing the peritoneal cavity after laparotomy irrigation with water at 110 deg. F. is advised. "When no tube is at hand, the water may be poured into the belly direct from the jug." It must be made to enter into the recesses of the pelvis and must be continued until it comes away clear. "Plain water that has been boiled and allowed to cool to the requisite temperature is the safest medium for peritoneal irrigation." The directions given by the authors may be thoroughly relied upon. Their teaching is evidently derived from their experience, and it is no small advantage that one of them at least is no mere specialist, but is also attached to a large general hospital.

The Analysis of Food and Drugs. Part I. By T. H. Pearmain and C. G. Moor, M.A. (Cantab.) (London: Baillière, Tindall, and Cox. 1897. 132 pages, 4 illustrations, index. Price 5s.)

This book is what it aims at being—a convenient book for laboratory use, containing all that is required for every-day routine work. But it is something more, viz., a book over which one might spend a most interested hour. It deals with the analysis of milk, cream, condensed milk, butter, and cheese. The natural variations in milk and their causes, its analysis, estimation of constituents, bacteriology, detection of adulterants, dyes, and preservatives, are thoroughly discussed and plainly expounded. Cheese and butter are dealt with in the same way. An analysis, highly useful to every medical practitioner, is given of the various sweetened or unsweetened, whole or separated, milks in the market. The very small number of these which are to be recommended as foods can be easily ascertained and kept in mind. A sample of condensed milk may, it is pointed out, be regarded as genuine in which the fat is true butter fat, and is not less than 10 per cent., the albuminoids not in excess of the fats, and the sample free from preservatives. The adulteration of butter by margarine is discussed. We learn that "at present we may purchase as 'cream cheese' what is not even a 'milk cheese.'" "Skimmed milk cheese containing only traces of fat may be sold as cheese without any qualification." Nevertheless, to sell such cheese adulterated with margarine is an offence against the law. A fair standard for cheese would be "not less than 30 per cent. of true butter fat, while no starch or other extraneous matter should be present." The authors point out that many of the forms of adulteration mentioned in the older text-books have disappeared, but they insist on the necessity for standards to which foods should conform. They believe that the quality of foods would by this means be "levelled up." The need of a proper standard for milk is a crying one. The limits adopted by the Society of Public Analysts and the Inland Revenue Department Laboratory are too low compared with the average composition of milk. Any milk falling below these limits should be regarded as adulterated. If so low a standard as this were made legal, the milk supply would be in danger of being brought to a dead level of inferiority. It is advisable to impose by law a limit as to the minimum amount of fat in all the products of the dairy, as well as the amount of water in bread and other foods. The book is a useful and very enjoyable one, and we look forward with pleasure to a second part.

